# Functional Aspects of Fibrin Structure Alterations by Tranexamic Acid in the Inhibition of Fibrinolysis

**DOI:** 10.3390/biom16050696

**Published:** 2026-05-08

**Authors:** Kata Balog Virag, Barbara Baráth, Kristóf Molnár, Petra Csikós, Alexandra Raska, László Szabó, Natalia Nikolova, Kiril Tenekedjiev, Krasimir Kolev, Nikolett Wohner

**Affiliations:** 1 Department of Biochemistry, Institute of Biochemistry and Molecular Biology, Semmelweis University, 1094 Budapest, Hungary; kalman.kata@semmelweis.hu (K.B.V.); barath.barbara@semmelweis.hu (B.B.); kristof0323@gmail.com (K.M.); cs.petra2000@gmail.com (P.C.);; 2 HCEMM-SU Thrombosis and Hemostasis Research Group, Department of Biochemistry, Semmelweis University, 1094 Budapest, Hungary; 3 Plasma Chemistry Research Group, Institute of Materials and Environmental Chemistry, Research Centre for Natural Sciences, 1117 Budapest, Hungary; 4 Department of Mechatronics, Faculty of Engineering, Nikola Vaptsarov Naval Academy, 9002 Varna, Bulgaria; natalianik@gmail.com; 5 Department of Computer Science, Varna Free University “Chernorizets Hrabar”, 9007 Varna, Bulgaria; ktenekedjiev@gmail.com; 6 Australian Maritime College, University of Tasmania, Launceston, TAS 7250, Australia

**Keywords:** fibrinolysis, tranexamic acid, prophylaxis, bleeding

## Abstract

Background: Tranexamic acid (TXA) is a synthetic lysine analog widely used as an antifibrinolytic agent. Large randomized trials have demonstrated life-saving benefits when TXA is administered early in acute hemorrhage, but results regarding prophylactic administration have been conflicting, and several trials have not shown improved clinical outcomes. The mechanisms underlying this discrepancy remain incompletely understood. Objectives: To investigate the molecular and structural mechanisms that determine TXA efficacy in purified fibrin clots under conditions mimicking therapeutic versus prophylactic administration. Methods: We examined fibrinolysis induced by tissue plasminogen activator (tPA) in vitro using confocal microscopy, viscoelastic testing (ClotPro), turbidimetry, and plasmin generation assays at physiologically and therapeutically relevant concentrations of plasminogen and TXA. Scanning electron microscopy (SEM) was employed to assess fibrin structure. Results: When TXA was incorporated into fibrin clots before the addition of tPA, physiological plasminogen concentrations (2.5 µM) reversed the antifibrinolytic effect, resulting in paradoxical acceleration of lysis. By contrast, when clotting and fibrinolysis occurred simultaneously in the presence of TXA and tPA, TXA consistently prolonged lysis time irrespective of plasminogen concentration. SEM demonstrated that TXA, even at concentrations as low as 16 µM, doubled the top-quartile values of the fibrin fiber diameter, altering susceptibility to plasmin-mediated degradation without accelerating plasminogen activation. Conclusions: TXA efficacy is determined not only by dose but also by timing and the plasminogen availability in the clot microenvironment. These findings provide mechanistic insight into the failure of prophylactic TXA administration and highlight the importance of context in optimizing its clinical use.

## 1. Introduction

Tranexamic acid (TXA) is a widely used antifibrinolytic agent with proven efficacy in the treatment of acute bleeding. Large randomized clinical trials have consistently demonstrated that early therapeutic administration of TXA can significantly reduce mortality from hemorrhage in trauma patients, as demonstrated in the CRASH-2 trial [1] and women with postpartum hemorrhage (WOMAN 1 trial) [2], as well as showing potential benefit in prehospital trauma settings, as shown in the STAAMP trial [3]. These findings have established TXA as a cornerstone of time-sensitive therapeutic intervention in bleeding emergencies.

In contrast, evidence on the prophylactic use of TXA has been inconsistent. Randomized trials in obstetrics have shown that prophylactic TXA does not significantly reduce the incidence of postpartum hemorrhage after vaginal birth [4], nor in women with anemia (WOMAN-2) [5]. Similarly, in patients with hematological malignancies and treatment-induced thrombocytopenia, large trials (A-TREAT; TREATT) demonstrated no meaningful reduction in bleeding complications or transfusion requirements with prophylactic TXA use [6,7]. Under surgical conditions, when the hemostatic and fibrinolytic factor levels are controlled, the prophylactic administration of TXA did not reduce postoperative bleeding in a cohort of bariatric patients [8].

In other settings, some data suggest that TXA may be effective when administered before overt bleeding occurs. In the large ATACAS trial, TXA was given after induction of anesthesia and before surgical incision in cardiac surgery, and this strategy markedly reduced perioperative blood loss, transfusion requirements, and the need for reoperation due to bleeding [9]. In the similarly large POISE-3 trial in non-cardiac surgery, TXA also significantly reduced major bleeding; however, in this study, the regimen (1 g at the start and 1 g at the end of surgery) cannot be considered purely prophylactic, as part of the dosing occurred immediately after the intervention [10].

These findings suggest that while TXA may reduce measured blood loss in some surgical settings, it does not reliably translate into improved clinical outcomes when used prophylactically.

Overall, current evidence highlights a striking difference between therapeutic and prophylactic applications of TXA. Whereas early therapeutic use in acute bleeding is life-saving, its routine prophylactic administration remains uncertain across multiple clinical contexts.

Although TXA is still classically regarded as a potent antifibrinolytic agent, accumulating evidence indicates that its effects are not always predictable. In addition to clinical observations, experimental studies have reported paradoxical alterations in fibrinolysis in the presence of TXA, including transient increases in plasmin generation and modulation of plasmin–antiplasmin complex formation [11]. Mechanistic investigations have further shown that lysine analogs such as TXA can induce an “open” conformation of plasminogen, thereby facilitating its activation under certain conditions [12]. Moreover, when urokinase plasminogen activator (uPA) predominates, TXA may even accelerate rather than inhibit fibrinolysis [13]. These observations, though less consistent than its well-established antifibrinolytic action, underscore that the net effect of TXA can be context-dependent and influenced by timing, dose, and the prevailing fibrinolytic pathway. Recently, it has also been reported that extremely high TXA concentrations reduce the angle and attenuate the rise in maximum amplitude in viscoelastic assays of plasma clots [14]. Issa and colleagues further demonstrated that such supraphysiological concentrations not only impair viscoelastic parameters but also directly alter fibrin architecture, increasing network density [14].

Based on the available experimental and clinical evidence, the mechanism of action of TXA appears to depend on the prevailing hemostatic state. Accordingly, the aim of the present study was to investigate the molecular and structural basis of the TXA effects in two in vitro experimental models: (1) lysis of fibrin clots with entrapped tPA at the stage of fibrin formation; (2) lysis of fibrin clots formed in the absence of tPA and subsequently attacked by extrinsic tPA. The former model mimics the efficacy of TXA in a clinical setting when it is administered to suppress ongoing bleeding accompanied by hyperfibrinolysis (therapeutic scenario), whereas the latter represents the situation in which prophylactic TXA is used, clots form in its presence and are subsequently exposed to tPA (prophylactic scenario).

## 2. Methods

### 2.1. Proteins and Reagents

Human fibrinogen (plasminogen-depleted) was obtained from Merck Life Science Kft., Budapest, Hungary. Bovine thrombin, purchased from Serva (Heidelberg, Germany), was further purified by ion-exchange chromatography on sulfopropyl-Sephadex, resulting in a preparation with a specific activity of 2100 IU/mg [15] and 1 IU/mL was considered equivalent to approximately 10.7 nM by active site titration [16]. Plasminogen and plasmin were prepared as previously described [17]. Recombinant tPA was from Boehringer Ingelheim, Ingelheim am Rhein, Germany. Tranexamic acid was obtained from Merck Life Science. Uncoated IBIDI VI 0.4 μ-slides were from Ibidi GmbH, Martinsried, Germany.

Alexa Fluor^®^ 546-conjugated fibrinogen from human plasma was from Invitrogen Life Technologies (Budapest, Hungary). Experiments were performed using HEPES-buffered saline (HBS: 10 mM HEPES buffer, pH 7.4, containing 150 mM NaCl) ± additives if not indicated otherwise.

### 2.2. Fibrinolytic Assays

#### 2.2.1. Confocal Microscopy of Fibrinolysis with Surface-Applied tPA (Extrinsic Fibrinolysis Model)

Fibrin clots were prepared at room temperature in sterile, uncoated IBIDI VI 0.4 μ-slides using 6 µM fibrinogen supplemented with varying concentrations of plasminogen (0.25, 0.75, or 2.5 μM). To allow fluorescence detection, 1% of the fibrinogen was replaced with Alexa Fluor^®^ 546-conjugated fibrinogen. Clots were formed in the presence or absence of TXA (128 μM) by adding 20 nM thrombin and incubating for 40 min. The concentration of thrombin and duration of the lytic assays were optimized for maximal sensitivity of the fibrin structure to inhibiting effects on lysis. Subsequently, 75 nM tPA was applied to the clot edge. Fluorescence at the clot–fluid interface (excitation 543 nm, emission 575 nm) was monitored using a confocal laser scanning microscope (LSM 510, Carl Zeiss GmbH, Jena, Germany). Sequential images of the fibrin–fluid boundary were acquired with a Plan-Neofluar 20×/0.5 objective, under identical laser intensities and exposure settings. The displacement of the lysis front was measured as a function of time.

#### 2.2.2. Viscoelastic ClotPro Assay (Fibrinolysis with tPA Incorporated in Fibrin)

Fibrinolysis in the presence of TXA was assessed using the ClotPro system (Enicor GmbH, München, Germany), a viscoelastic device that continuously monitors clot formation and stability under dynamic conditions. During clotting, fibrin adheres to the cup and pin surfaces, enabling continuous measurement of clot mechanical strength [18]. While point-of-care testing commonly employs pre-filled active tips, we developed an in-house assay incorporating 2 nM tPA. The assay used fibrinogen at 6 µM supplemented with plasminogen at 0.25, 0.75, and 2.5 μM, with TXA applied at varying concentrations in the range 0–128 μM (0–20 mg/L, with the upper limit of the concentrations used in our experiments corresponding to a therapeutically relevant TXA concentration in human plasma) [19], and clotting was initiated with 80 nM thrombin in a final volume of 340 μL. For analysis, maximal clot firmness (MCF, the maximum amplitude of the viscoelastic trace, reflecting the highest mechanical strength and stability of the clot during measurement) and lysis time (LT) were used. LT was defined as the interval between clotting time (CT; time to 2 mm amplitude) and the point at which 50% of MCF was lost. LT values were provided by the ClotPro Analytical Software Version 1.45a.

### 2.3. Turbidimetry (Fibrinolysis with tPA Incorporated in Fibrin)

Fibrin formation and dissolution were monitored by measuring the light absorbance at 340 nm at 37 °C with a CLARIOstar spectrophotometer (BMG Labtech, Ortenberg, Germany). Clots were prepared from 6 µM fibrinogen containing plasminogen (0.25, 0.75, or 2.5 µM), and 0.15 nM tPA. Tranexamic acid at 0–128 µM was added where indicated. Coagulation was initiated with 15 nM thrombin, and turbidity curves were recorded continuously.

### 2.4. Plasminogen Activation Assay

In 96-well microtiter plates, fibrinogen at 6 µM supplemented with various concentrations of plasminogen (0.25, 0.75, or 2.5 µM), and TXA in the range 0–128 μM were clotted with 80 nM thrombin for 50 min in a total volume of 80 µL. Subsequently, a mixture of 2 nM tPA and 250 μM fluorescent plasmin substrate Boc-Glu-Lys-Lys-AMC (4-methylcoumaryl-7-amide, Bachem AG, Bubendorf, Switzerland) in 60 µL was layered onto the clot surface, restricting plasminogen activation to a thin interfacial layer, as previously described for this type of two-phase assay [20]. Fluorescence (excitation 360 nm, emission 450 nm) was continuously recorded using a Clariostar fluorophotometer (reflecting the release of AMC) by plasmin for periods when less than 10% of the fluorogenic substrate was consumed. The rate of plasminogen activation was quantified as the slope of the linear function relating fluorescence to time squared, as introduced previously for chromogenic assays [21]. Independent experiments (unique combinations of plasminogen and TXA), each in 5 replicates, were analyzed. The relative efficiency of plasminogen activation was estimated from the ratio of the experimental slopes measured in the presence of TXA and its absence at each of the 3 plasminogen concentrations.

### 2.5. Morphometric Analysis of Fibrin Structure

Fibrinogen (at 6 µM) was supplemented with TXA in the range 0–128 μM and clotted with 80 nM thrombin for 120 min at 37 °C. Fibrin clots of 50 μL volume were washed with 10 mL 100 mM Na-cacodylate pH 7.2 buffer, and samples were fixed in 1% *v*/*v* glutaraldehyde for 16 h. The fixed samples were dehydrated in a series of ethanol dilutions (20–96% *v*/*v*), a 1:1 mixture of 96% *v*/*v* ethanol/acetone, and pure acetone, followed by critical point drying with CO_2_ in the E3000 Critical Point Drying Apparatus (Quorum Technologies, Newhaven, UK). The specimens were mounted on adhesive carbon disks, sputter-coated with gold in SC7620 Sputter Coater (Quorum Technologies, Newhaven, UK), and images were taken with scanning electron microscope EVO40 (Carl Zeiss GmbH, Oberkochen, Germany). The diameter of 400 fibers on each of 5 independent clot images was measured by placing the pointer of the Distance tool of the Image Processing Toolbox v. 11.7 of Matlab 2023a (The Mathworks, Natick, MA, USA) over the endpoints of transverse fiber cross-sections.

### 2.6. Statistical Analysis

The effects of TXA on the fibrinolytic (confocal microscopy, turbidimetry, and viscoelasticity) assay parameters were analyzed with the Mann–Whitney U test for differences between TXA-treated and untreated groups, with *p* < 0.05 considered significant. A subgroup analysis was performed within the turbidimetry datasets to compare the Amax values of the TXA-free groups across different plasminogen concentrations using the Kruskal–Wallis test. These tests were run under GraphPad Prism ver. 10.6.1 (GraphPad Software, LLC, San Diego, CA, USA).

For the statistical evaluation of the relative efficiency of plasminogen activation, the ratios of the experimental slopes measured in the presence and absence of TXA were treated as random variables, with the numerator and denominator measured with certain experimental error. A total of 10,000 Bootstrap pseudo-realities (pairs of synthetic samples of the ratios) were created by drawing with replacement from the experimental datasets [22]. The *p*-values of 7 Bootstrap statistical tests were calculated: Kuiper Bootstrap test for distribution equality [23], two-tailed and one-tailed Bootstrap test for median equality [24], two-tailed and one-tailed Bootstrap test for bottom quartile equality, two-tailed and one-tailed Bootstrap test for top quartile equality [25]. The test statistics for the respective Bootstrap hypothesis tests were as follows: Kuiper’s statistic for the distribution test, the difference in medians for the median test, and the difference between the bottom and top quartiles for the bottom and top quartile tests, respectively. The applied approach for assessing differences between two random variables using two crisp samples addresses the debate over *p*-value validity [26]. By using a cluster of tests instead of a single *p*-value, our conclusions gain additional statistical validity, and the likelihood of false-positive findings is substantially minimized, as previously used [24,25].

For statistical evaluation of the distributions of fibrin fiber diameter, datasets from each image were compared to identify any outlier datasets. A dataset was considered as outlier, if all of the following three conditions were valid: (1) its probability to be greater than a value from at least two other datasets was out of the range 0.25–0.75; (2) the Kuiper’s test rejected the hypothesis for distribution equality with at least two other datasets; (3) the Bootstrap statistical tests rejected the hypothesis for equality of at least three out of four numeric characteristics (mean, median, bottom and top quartile) with at least two other distributions at probability level *p* < 0.05. Following this outlier analysis, which resulted in the exclusion of two out of the twenty evaluated samples, the datasets from the same type of fibrin clots were merged, the best-fitting theoretical distribution of fiber size was determined from 10,000 Bootstrap replicas, and the fitted distributions were compared using Kuiper’s test, as described previously [25]. The Bootstrap and Kuiper’s tests were performed with the Statistics and Machine Learning Toolbox v. 24.2 of Matlab.

## 3. Results

### 3.1. Tranexamic Acid Is Pro-Fibrinolytic in an Extrinsic Lysis Assay If It Is Incorporated in the Structure of Fibrin Containing Physiological Levels of Plasminogen

**Figure 1:**
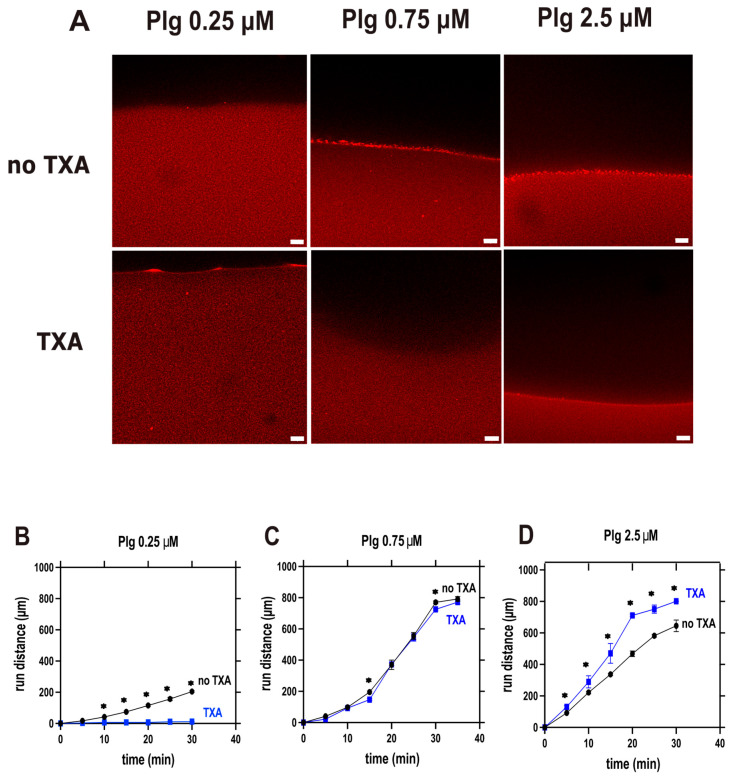
The antifibrinolytic effect of tranexamic acid incorporated in fibrin is reversed by high plasminogen levels. Fibrin clots were generated from fibrinogen (6 µM, 1% Alexa-546-labeled) supplemented with plasminogen at the indicated concentrations and clotted with thrombin, in the absence or presence of tranexamic acid (TXA, 128 μM). After 40 min, tPA (75 nM) was applied to the clot surface to initiate lysis. Fluorescence of the fibrin (excitation 543 nm, emission 575 nm) was monitored by confocal laser-scanning microscopy. (**A**) Representative images taken at 20 min lysis of clots with the indicated composition (scale bar: 50 μm). (**B**–**D**) Time course of the displacement of the fibrin boundary layer as fibrin is resolved. Median (bottom/top quartile) values are shown. Asterisks indicate significant differences between the lysis in the absence and presence of TXA according to the Mann–Whitney U test (*p* < 0.05, *n* = 4).

Incorporating TXA into fibrin clots prior to lysis onset provides an in vitro model of fibrin susceptibility to fibrinolysis under prophylactic TXA conditions. When we monitored the lysis of fibrin by tPA applied to the surface of the clot (extrinsic fibrinolysis) using confocal microscopy, pre-incorporated TXA substantially modified the degradation dynamics, and in certain constellations, its effect deviated from the classical antifibrinolytic profile typically assumed for this agent (Figure 1).

The impact of TXA on fibrinolysis in this extrinsic fibrinolysis setting was strongly dependent on plasminogen concentration (Figure 1). At low plasminogen levels, TXA markedly inhibited clot breakdown, consistent with its established antifibrinolytic effect (Figure 1B,C). As plasminogen concentration increased, reaching its physiological levels in circulation [27], this inhibitory action was progressively attenuated, resulting in negligible effects at intermediate levels. At physiological plasminogen concentrations, TXA no longer delayed fibrinolysis but, on the contrary, accelerated clot lysis, indicating a paradoxical, concentration-dependent effect of the drug on global fibrinolysis (Figure 1D).

### 3.2. Consistent Anti-Fibrinolytic Effects of TXA in Intrinsic Fibrinolytic Assays

**Figure 2:**
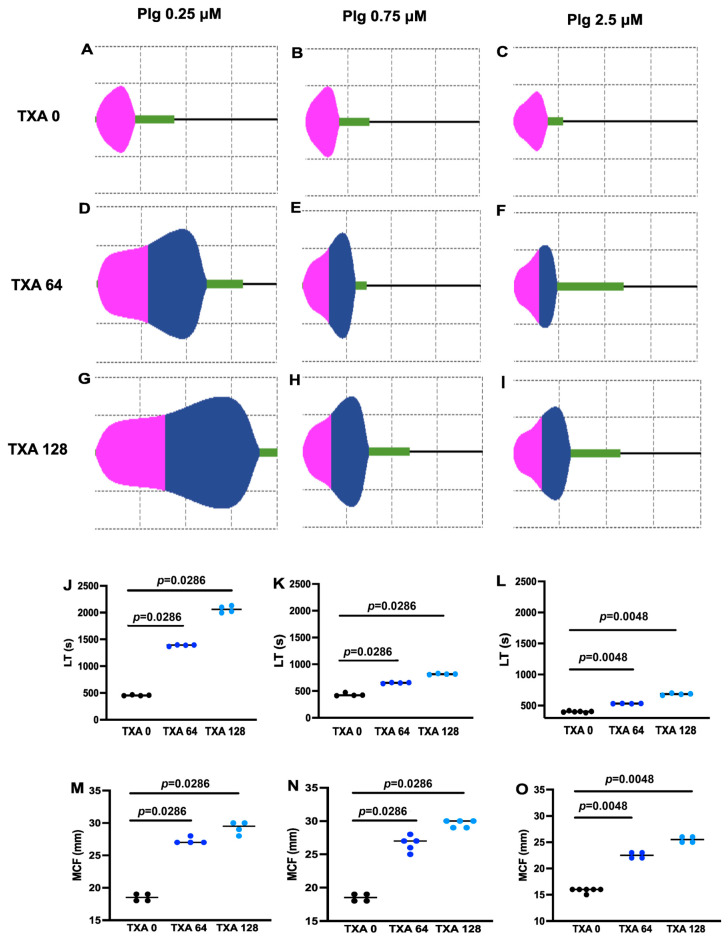
TXA consistently prolongs lysis time (LT) and increases maximal clot firmness (MCF) in a thromboelastographic assay of intrinsic fibrinolysis. Fibrin clots were prepared from fibrinogen at 6 µM supplemented with plasminogen at the indicated concentrations, tPA at 2 nM, and TXA (0, 64, 128 µM) and clotted with thrombin (80 nM). (**A**–**I**) Representative thromboelastograms for the indicated experimental conditions, on which one major grid square corresponds to 10 mm vertically (MCF) and 10 min horizontally (time). (**J**–**O**) Lines indicate the median values LT (**J**–**L**) and MCF (**M**–**O**) as quantitative parameters of intrinsic fibrinolysis, symbols indicate the individual measured samples. The *p*-values for the pairs of samples linked by lines are shown according to the Mann–Whitney U test.

**Figure 3:**
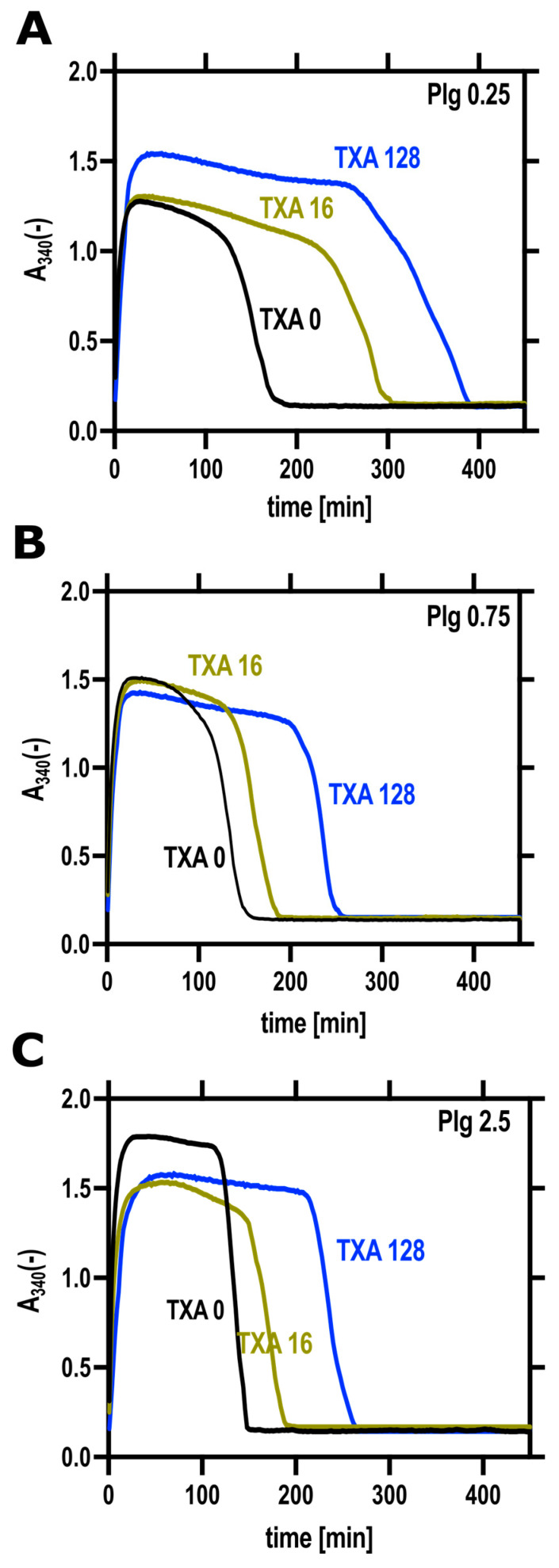
TXA consistently prolongs the lysis time in a turbidimetric assay of intrinsic fibrinolysis, whereas its effect on maximal turbidity depends on the plasminogen–TXA balance. Clots were formed from fibrinogen at 6 µM supplemented with plasminogen at 0.25 µM (**A**), 0.75 µM (**B**), or 2.5 (**C**), tPA at 0.15 nM, and TXA (0, 16, 128 µM), and clotted with thrombin at 15 nM. Absorbance at 340 nm was recorded continuously at 37 °C with at least 4 independent replicates per condition. Amax was defined as the maximum absorbance during clot formation. We measured Amax and the time from Amax to 50% of Amax (lysis time, LT50) and the numeric results are shown in Table 1.

The acceleration of fibrinolysis observed at high plasminogen-to-TXA ratios was not reproduced in conventional intrinsic fibrinolytic assays, in which tPA was incorporated into the clot under otherwise identical experimental conditions (Figure 2 and Figure 3). In the viscoelastic ClotPro test, TXA (64–128 µM) consistently prolonged lysis time irrespective of plasminogen concentration (Figure 2J–L). Notably, across all experimental settings of the viscoelastic assay, TXA increased the maximal clot firmness (MCF) in parallel with the prolongation of lysis time (Figure 2M–O).

In turbidimetric assays, TXA consistently prolonged clot lysis times (LT50) across both low and physiological plasminogen concentrations (Figure 3, Table 1). Changes in maximum absorbance (Amax), however, were less coherent. Amax significantly increased with rising concentrations of plasminogen. This increase in Amax at high plasminogen concentrations could be attributed to effects reported in previous studies, which indicate that the behavior of Amax in such intrinsic fibrinolytic assays is complicated by frequently observed transient increases in turbidity during lysis [28] and that some peptides corresponding to portions of fibrin(ogen) can increase turbidity [29]. Such effects are more probable at accelerated lytic rates with high plasminogen concentrations. However, TXA at the highest concentration of 128 µM applied in our assay efficiently moderated them and generated clots of similar turbidity independently of the plasminogen concentration (Table 1).

### 3.3. Acceleration of Extrinsic Fibrinolysis by TXA Is Independent of the Plasminogen Activation Phase

**Figure 4:**
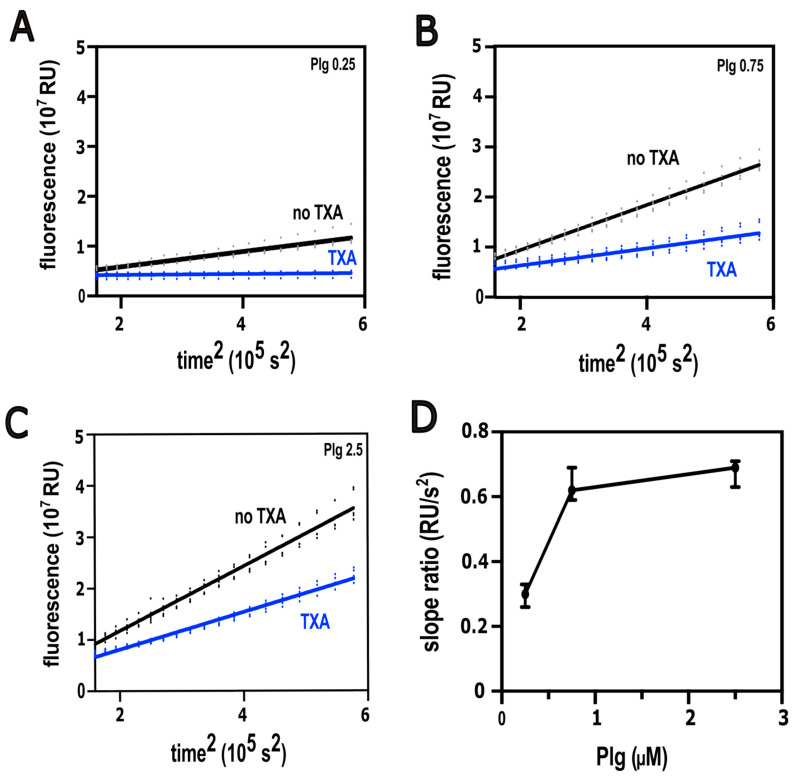
Combined effects of TXA and plasminogen on plasminogen activation by tPA on the surface of fibrin clots. Fibrinogen containing plasminogen and TXA at 128 μM was clotted with thrombin, and after clotting, a mix of 2 nM tPA and 250 μM plasmin substrate Boc-Glu-Lys-Lys-AMC was layered on the fibrin surface. The fluorescence at 360 nm excitation and 450 nm emission was continuously measured, indicating the release of AMC (4-methylcoumaryl-7-amide) by the generated plasmin (representative raw fluorescence data are shown in (**A**–**C**) for the plasminogen concentrations indicated in µM). The velocity of plasminogen activation was calculated as the slope of the linear function of fluorescence versus time-squared, as derived in [21] for chromogenic assay of plasminogen activation. The slopes of at least 3 to 10 independent measurements were used to generate 10,000 bootstrap-simulated velocity ratios for comparison with different baseline reference values, the bias-corrected median values of which (top-bottom quartiles) are shown in (**D**).

Given the divergent effects of TXA incorporated in fibrin on lysis by extrinsically and intrinsically applied tPA, a plausible mechanism for accelerated extrinsic fibrinolysis could be the enhancement of plasmin generation on the surface of pre-formed clots. To test this hypothesis, we used a plasminogen activation assay, in which a fluorogenic plasmin substrate is layered together with tPA onto the surface of pre-formed fibrin clots. Thereby, the plasmin generated at the clot/fluid interface is measured (Figure 4A–C). The slope of the fluorescence signal plotted against time-squared provided a direct measure of the plasminogen activation rate [21,30]. The ratio of activation velocities in the presence versus absence of TXA was used to characterize its modulatory effect. TXA inhibited the generation of plasmin at all concentrations of plasminogen in the clot. The degree of inhibition was moderated at higher plasminogen concentrations (Figure 4D), but no enhancement was seen for the plasminogen concentration promoting lysis in the extrinsic setup of Figure 1. Therefore, the role of enhanced plasminogen activation could be excluded as a mechanism of acceleration of extrinsic fibrinolysis.

### 3.4. Tranexamic Acid Induces Substantial Structural Modifications in Fibrin Architecture

**Figure 5:**
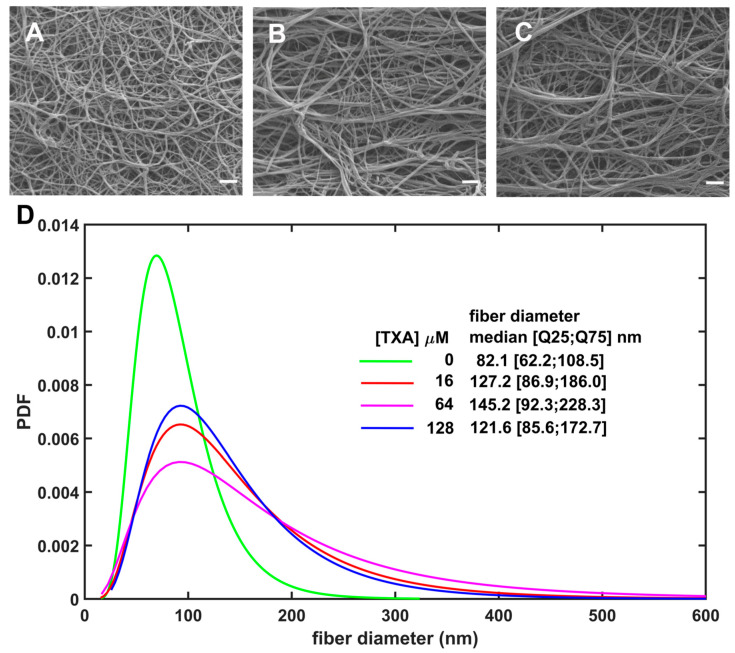
TXA increases the fiber diameter of fibrin. Fibrinogen (at 6 μM) was supplemented with TXA (0; 16; 64; 128 μM) and clotted with 80 nM thrombin for 120 min at 37 °C. Fibrin fiber diameters were measured as described in the Methods section. For each condition, the diameters of 400 fibers were measured from 5 independent images. (**A**–**C**) Representative scanning electron microscopy images of fibrin clots containing 0 (**A**), 16 (**B**) or 64 (**C**) μM TXA (scale bar: 1 μm). (**D**) Probability density functions (PDFs) of the best-fitting theoretical distribution of fiber diameters derived from 10,000 synthetic bootstrap replicas drawn with replacement from the measured datasets after outlier exclusion, as detailed in the Methods section. Median [bottom Q25, top Q75 quartiles] of the fitted diameter distributions are shown. For all pairwise comparisons, Kuiper bootstrap tests indicated significant differences in distribution (*p* < 0.001).

Since fibrin network architecture influences both stages of interfacial fibrinolysis [31] (1) plasminogen activation (by providing a scaffold for tPA–plasminogen complex assembly) and (2) fibrin degradation (as the substrate of plasmin), we examined the structure of fibrin clots by scanning electron microscopy (Figure 5).

All tested TXA concentrations, including lower than the peak of clinically relevant levels, caused a significant thickening of fibrin fibers seen as a shift to the right in the probability distribution of fiber size (Figure 5D). The most prominent effect of TXA was the more than 2-fold shift in the fiber diameter in the top 25% (Q75) of the thickest fibers from 108.5 nm in the absence of TXA to 228.3 nm at 64 µM TXA (Figure 5D). Based on this data, a rough estimate of the TXA level producing half maximal effect would be a concentration lower than 16 µM TXA, the lowest one applied in our SEM measurements.

## 4. Discussion

We found that TXA, a synthetic lysine analog widely used for its antifibrinolytic properties, exhibits context-dependent efficacy in fibrinolysis, which may account for its seemingly contradictory clinical behavior. Timing emerges as a critical determinant of TXA effectiveness. Consistent with clinical observations showing limited benefit when TXA is administered too late in bleeding complications, prophylactic administration has also often proven ineffective—an outcome that remains incompletely understood. Our findings provide a mechanistic explanation, demonstrating that the action of TXA depends not only on the timing of its incorporation into the emerging clot but also on the characteristics of the clot microenvironment, particularly the balance between plasminogen concentration and TXA availability within the fibrin network.

By competitively binding to the lysine-binding sites of plasminogen and plasmin, TXA prevents their interaction with fibrin, thereby inhibiting fibrin-surface–dependent activation of plasminogen by tissue plasminogen activator (tPA) and stabilizing clot structure [32]. This mechanism has been extensively validated in biochemical studies and underpins the clinical use of TXA in trauma, surgery, and obstetrics to reduce bleeding [32]. However, clinical and laboratory observations sometimes reveal paradoxical responses. Mechanistic studies have provided explanations for these paradoxical findings. One of the earliest demonstrations was that lysine analogs such as TXA can induce an “open” conformation of plasminogen in solution, making it more readily activated [12]. Sharma et al. reported decreased clot firmness and prolonged clotting time in patients receiving TXA during cardiac surgery [13].

Other investigators have also highlighted that TXA’s effects can be more complex and condition-dependent. For instance, Longstaff et al. discuss scenarios in which elevated urokinase plasminogen activator (uPA) activity diminishes the efficacy of TXA, or even allows residual fibrinolysis despite blockade of tPA pathways. This supports the idea that uPA-driven fibrin or non-fibrin proteolysis may contribute when TXA concentrations or timing allow [33]. TXA can also protect free plasmin from inhibition by α_2_-antiplasmin at higher concentrations [11,34]. In addition, ε-aminocaproic acid, a less effective analog of TXA, has been shown to facilitate plasmin diffusion within fibrin by reducing its binding to C-terminal lysines, thereby accelerating clot degradation in experimental models [35,36]. The impairment of the tPA binding to fibrin by TXA is also expected to facilitate the tPA diffusion into the clot, as observed with reteplase, a mutant tPA variant lacking essential fibrin-binding domains [37,38].

These mechanisms provide a plausible explanation for the failure of TXA to prevent bleeding when administered more than 3 h after trauma, a clinical scenario where uPA activity becomes dominant as tPA levels decline. Indeed, in a murine traumatic brain injury model, TXA administration during the late phase promoted fibrinolysis when uPA was the principal plasminogen activator [39]. Collectively, these data suggest that while TXA is strongly antifibrinolytic in the early phase dominated by tPA activity, later conditions may unmask pro-fibrinolytic actions, underscoring the importance of timing and activator pathway in determining its net effect.

Our current findings suggest a new aspect of the efficiency of early TXA administration [40] when conventional electrolyte replacement therapy of major bleeding causes dilution of circulating plasminogen to concentrations at which we observe improved anti-fibrinolytic effects of TXA. Conversely, at physiological concentration, plasminogen competes with TXA using the same binding sites, which can at least explain a loss of anti-fibrinolytic effectiveness of TXA at higher plasminogen concentration, as observed in our current study. We assessed the effect of clinically relevant TXA concentrations [41], including those at the upper end of the therapeutic range, on fibrinolysis inhibition under plasminogen concentrations that likewise reflect physiological levels [31]. Previous studies indicate that substantial inhibition of fibrinolysis occurs at plasma TXA concentrations of approximately 60–95 µM, while concentrations in the range of 30–60 µM may already exert partial antifibrinolytic effects [19]. In our experimental settings, we used TXA concentrations ranging from 0 to 128 µM, which are within the therapeutically relevant plasma range in humans and remain below the peak concentrations reported in plasma after intravenous TXA administration [19]. In global fibrinolysis assays, we compared two conditions intended to model prophylactic and therapeutic TXA administration in a simplified experimental system: clots in which TXA was incorporated before the addition of tPA to the surface (Figure 1), and clots formed in the simultaneous presence of both TXA and tPA, where clotting and lysis occurred dynamically (thromboelastography and turbidimetry, Figure 2 and Figure 3). In the former setup, at higher plasminogen concentrations, TXA unexpectedly enhanced lysis, whereas this effect was never observed in the intrinsic lysis assays. This finding suggests that an important structural modification occurs when clot formation takes place in the presence of TXA—a change that does not arise if tPA is present from the outset and initiates lysis immediately. Using SEM, we confirmed this structural effect (Figure 5), showing that TXA, even at the lowest tested concentration (16 µM), significantly increased fibrin fiber thickness. Compacting identical amounts of fibrin monomers in thicker fibers also implies larger pores in the fibrin meshwork. The thicker fibers are known to be a better substrate for plasmin, whereas the larger pores allow deeper penetration of plasmin if it is generated on the clot surface (reviewed in Weisel et al. [42]). These alterations of the fibrin structure have less impact on plasminogen activation if it is examined independently of fibrin degradation, as demonstrated in our plasminogen activation assay; however, other structure-dependent mechanisms, including altered tPA distribution, fibrin-binding kinetics, and plasmin diffusion, may also contribute importantly to the modification of TXA effects within the clot [43,44].

In a recent study, Issa et al. demonstrated that TXA at high concentrations can directly modify fibrin architecture, with 10–20 mg/mL (≈64–128 mM) concentrations producing a marked increase in fibrin network density, whereas lower concentrations (2.5 mg/mL, ≈15 mM) had little effect on network structure [14]. It is important to note, however, that these concentrations are extremely supraphysiological; in clinical practice, peak plasma levels of TXA typically reach only 500–1000 µM (≤1 mM) [32,45], which is one to two orders of magnitude lower than the concentrations examined by Issa et al.

The same study also demonstrated that TXA exerts concentration-dependent effects on clot viscoelastic properties: at 15 mM, TXA increased the clot angle and maximum amplitude—changes consistent with antifibrinolytic activity, whereas at 150 mM, TXA reduced the angle and attenuated the rise in maximum amplitude. In contrast, and importantly distinct from the non-physiological concentrations applied in the cited study, our findings indicate that clot dynamics are not determined by TXA concentration alone. Rather, the plasminogen/TXA ratio within the clot microenvironment governs whether clot characteristics are modified, both at low and high TXA levels (remaining in the physiological range).

According to our interpretation, early incorporation of TXA into small clots formed at sites of microvascular injury may render them more susceptible to lysis. As a result, its prophylactic effect is less pronounced than when administered concurrently with bleeding, when substantial amounts of tPA are already released from the injured vessel wall. This represents a mechanism distinct from that proposed for delayed administration, where accelerated uPA-mediated plasminogen activation further complicates the overall scenario.

A major limitation of this study is that our experiments were performed in purified static systems that do not recapitulate the complexity of physiologic clot formation and lysis. Accordingly, the translational interpretation of the partly contradictory effects of TXA should be interpreted with caution. Our model lacked cellular elements and platelets, FXIII-mediated fibrin and α2-antiplasmin crosslinking, TAFI activity, and flow, each of which can substantially modify fibrin structure and fibrinolytic resistance. Available evidence indicates that red blood cells suppress fibrinolysis and act synergistically with TXA to enhance antifibrinolytic activity [25]. Factor XIII appears to further support this effect by increasing red blood cell retention within contracting clots, thereby contributing to clot stabilization [46]. Platelets can promote thrombin generation, alter fibrin ultrastructure, and provide antifibrinolytic mediators such as PAI-1 and TAFI [47,48]. FXIII/α2-antiplasmin system stabilizes fibrin against lysis, with incorporation of α2-antiplasmin into fibrin being a major determinant of clot resistance to fibrinolysis [49,50]. Since TXA modulates fibrin-dependent plasminogen/plasmin interactions, its apparent effect in our simplified system may differ from that in a mature clot in which α2-antiplasmin is incorporated into the fibrin network and dynamically regulates local plasmin activity [51]. TAFI limits fibrinolysis by removing C-terminal lysine residues from fibrin, thereby reducing plasminogen and tPA binding, a mechanism that is particularly relevant in the context of TXA, which also targets lysine-dependent fibrinolytic interactions [52]. Thus, in a TAFI-competent system, the overall effect of TXA may be quantitatively or even qualitatively different from that observed here, depending on the extent to which these two antifibrinolytic mechanisms act redundantly or synergistically. Finally, flow shapes thrombus composition and fibrinolytic transport, and high-shear thrombi may be relatively more resistant to lysis because of altered local distribution of tPA, plasminogen, and PAI-1 [53,54]. Therefore, our findings should be regarded as mechanistic rather than directly translational, and should be validated in more physiological models.

## 5. Conclusions

In summary, our data provide new insights into the mechanisms by which TXA may lose efficacy in a time-dependent manner and potentially also in the prophylactic setting. Further studies are required to precisely define the optimal dose, the ideal timing of administration, and the patient populations most likely to derive benefit from this therapy.

## Figures and Tables

**Table 1:** Quantitative parameters of intrinsic fibrinolysis in the presence of TXA measured in a turbidimetric assay.

Plg (μM)	TXA (μM)	A_max_(-)	LT50 (min)
0.25	0	1.264 **^†^** (1.298;1.253)	148.4 (156.7;144.2)
	16	1.283 (1.347;1.249)	303.4 * (275.7;248)
	128	1.505 * (1.539;1.456)	338.3 * (346;333.8)
0.75	0	1.502 **^†^** (1.545;1.490)	128.8 (134.4;124.7)
	16	1.486 (1.585;1.426)	160.4 * (170.1;156.2)
	128	1.431 * (1.435;1.417)	237.8 * (238.6;236.9)
2.5	0	1.78 **^†^** (1.82;1.76)	134.2 (135.4;133.7)
	16	1.50 (1.73;1.44)	155.1 * (160;149)
	128	1.585 * (1.59;1.581)	222.9 * (234.2;219.8)

The peak absorbance during clot formation (Amax) and from the time after Amax to reach 50% of Amax (lysis time, LT50) were determined as described in Figure 3. Data are presented as median with interquartile range. An asterisk and bold font indicate statistical significance according to the Mann–Whitney U test (n = 4) compared with the corresponding TXA-free samples. Dagger indicates statistical significance based on the Kruskal–Wallis test for TXA-free samples across three plasminogen concentrations.

## Data Availability

The original contributions presented in this study are included in the article. Further inquiries can be directed to the corresponding author.
